# *Apln-CreERT:mT/mG* reporter mice as a tool for sprouting angiogenesis study

**DOI:** 10.1186/s12886-017-0556-6

**Published:** 2017-09-02

**Authors:** Jingjiang Pi, Yu Cheng, Huimin Sun, Xiaoli Chen, Tao Zhuang, Jie Liu, Yixi Li, Huan Chang, Lin Zhang, YuZhen Zhang, Ting Tao

**Affiliations:** 1 Key Laboratory of Arrhythmias of the Ministry of Education of China, Research Center for Translational Medicine, Shanghai East Hospital, Tongji University School of Medicine, Shanghai, 200120 China; 2 Department of Ophthalmology, Ruijin Hospital, Shanghai Jiaotong University, School of Medicine, 197 Ruijin Er Rd, Huangpu District, Shanghai, 200025 China; 3 Dalian Medical University, Liaoning, 116044 China; 4 Department of Geriatrics, Ruijin Hospital, Shanghai Jiaotong University, School of Medicine, 197 Ruijin Er Rd, Huangpu District, Shanghai, 200025 China

**Keywords:** Retina, Sprouting angiogenesis, Membrane tomato red (mT), Membrane GFP (mG), Apelin

## Abstract

**Background:**

Angiogenesis is defined as a new blood vessel sprouting from pre-existing vessels, and the sprouting angiogenesis is the start phase of angiogenesis, which is critical for both physiological and pathological processes, such as embryonic development, organ growth, wound healing, tumor growth, diabetic retinopathy and age-related macular degeneration. Better understanding of the mechanisms of sprout angiogenesis will provide a rationale for the treatments of these angiogenesis related diseases.

**Methods:**

*mT/mG* tool mice are crossed with *Apln-CreERT* mice to generate *Apln-CreERT: mT/mG* mice, then we used neonatal retinal angiogenesis model to observe the angiogenic pattern of *Apln-CreERT:mT/mG* mice compared with *Cdh5-CreERT:mT/*mG mice. FACS analysis was used to sort eGFP and tdTomato endothelial cells (ECs) for measuring *Apelin* and *Cdh5* expression. Retinal sprouting angiogenesis pattern was also observed at different neonatal time when induced by tamoxifen and at hypoxia condition, as well as in vivo tumor in real-time angiogenesis in a dorsal skinfold window chamber in *Apln-CreERT:mT/mG* mice.

**Results:**

*Apln-CreERT:mT/mG* mice exhibited eGFP signal only in the sprouting angiogenesis, with less eGFP expression in the retinal “optic nerve” area than in that of *Cdh5-CreERT: mT/mG* mice, which might be due to relative mature vessels in the “optic nerve” area. The ECs sorted by FACS confirmed that the *Apelin* expression level was higher in eGFP ECs than tdTomato ECs of “optic nerve” area. Further we found that GFP-labeled sprouting angiogenesis decreased gradually following tamoxifen administration from P5-P7, but increased significantly during hypoxia in *Apln-CreERT:mT/mG* mice. At last, using *Apln-CreERT:mT/mG* mice we found tumor sprouting angiogenesis in dorsal skinfold, but not in the normal skinfold tissue.

**Conclusions:**

*Apln-CreERT:mT/mG* mouse line is a useful tool to differentiate sprouting angiogenesis from whole blood vessels in the investigation of retinal and tumor sprouting angiogenesis in vivo*.*

## Background

Angiogenesis is defined as a new blood vessel sprouting from pre-existing vessels. Sprouting angiogenesis is a critical process for both physiological and pathological processes, such as embryonic development, organ growth, wound healing, tumor growth, diabetic retinopathy and age-related macular degeneration and rheumatoid arthritis [1, 2]. This highly regulated process takes place through two non-exclusive events, the so-called endothelial sprouting or non-sprouting microvascular growth. In sprouting part, endothelial cells (ECs) can be defined as tip cells, stalk cells, and phalanx cells. Vital pathways, such as Notch and Notch ligands, VEGF and VEGFRs, Semaphorins and Netrins, take part in this process. Beyond that, ECs belonging to angiogenic sprouting will determine the growth speed and direction of angiogenesis [3]. The superficial vascular plexus forms during the first week after birth by radial outgrowth of vessels from the optic nerve into the periphery, reaching the retinal edges at approximately P8, from P7 onward the superficial capillaries start sprouting vertically to form first the deep and then the intermediate vascular plexus [4]. Apelin (Apln) has been confirmed to play an important role in sprouting, which are abundant in sprouting angiogenesis [5, 6].

Cre-mediated recombination of *loxP* is one of the most widely used genetic tools to study in vivo cellular and molecular mechanisms. Cre-mediated recombination can induce tissue-specific gene gain- or loss-of-function based on *loxP* sites in conditional overexpression gain-of-function or conditional knockout loss-of-function mouse line. Depending on the responding allele, Cre recombinase can either knockout a gene by removing intervening coding sequence flanked by the floxed *loxP* sites, or activate a gene by excising upstream floxed transcriptional STOP cassettes. Cre-*loxP*-mediated recombination also enables in vivo lineage tracing when used in conjunction with a reporter allele that expresses an indelible marker following excision of STOP cassette, and Cre-*loxP* system have been invented to study gene function of specific tissue or cell depending on the promoter to drive the Cre expression [7–9].

The membrane (m) fluorescent *mT/mG* reporter mice (Jackson strain: B6.129(Cg)-Gt (ROSA)26Sortm4(ACTB-tdTomato, −EGFP) Luo/J) contain a single copy of the transgene integrated into the ROSA locus. The transgene cassette is comprised of a chimeric CMV, β-actin promoter driving the expression of a floxed membrane localized Tomato tandem dimer. Following Cre-mediated excision of the stop codon, the membrane tdTomato transgene is removed, and the CAG promoter drives expression of membrane localized eGFP. In this study we crossed the *mT/mG* reporter mice with transgenic mice expressing a tamoxifen regulated *Apelin* promoter driven Cre recombinase for the study of sprouting angiogenesis, or a tamoxifen regulated *Cdh5* promoter driven Cre recombinase for the study of whole angiogenesis [10].

Hypoxia is one of the most potent inducers of sprout angiogenesis, which stimulates vascular invasion and growth into oxygen- and nutrient-deficient tissues. The master regulators of hypoxia-induced gene expression are the transcription factors of hypoxia-inducible factor (HIF) family. Under hypoxic conditions, HIF1α induces the expression of several pro-angiogenic molecules. One of these molecules is Apelin [11]. Thus, we observed the retinal sprouting angiogenesis under hypoxia condition and compared with normoxia condition in *Apln-CreERT:mT/mG* mice.

Successful angiogenic restriction for cancer therapy requires strategies not only the effects on tumor growth but also on endothelial tip cell sprouting, vascular maturation and recruitment of endothelial progenitor cells [12]. *Apln-CreERT* mouse line has been generated for studying tumor sprout angiogenesis [13, 14], but the application of this mouse line on retina sprouting angiogenesis has not been reported before. Furthermore, applying this mouse line to observe the tumor real-time angiogenesis has not been studied before. Therefore, *Apln-CreERT* mouse line was used to observe the retinal developmental sprouting angiogenesis, as well as the tumor in vivo in real-time sprouting angiogenesis by the dorsal skinfold window chamber model, trying to provide opportunities for further detailed mechanism study of sprouting angiogenesis and find therapeutic target for intervention of tumor angiogenesis to impair the tumor growth.

## Methods

### Animal

R26R-tdTomato-eGFP line (mT/mG, JaxMice, stock number 007576) was used as reporter to show the green-eGFP expressing blood vessels when crossing with *Cdh5* or *Apln* promoter-driven Cre mouse line [14–16]. Mice were maintained in stable temperature (22 ± 2 °C), humidity (55 ± 5%) and controlled illumination (12/12 h light/dark cycle) and under non-pathogenic conditions. Animals were sacrificed using isoflurane followed by cervical dislocation.

### Mouse retina angiogenesis model

A well-established mouse retinal developmental angiogenesis model was used to observe the cell specific effects of Apelin on angiogenic blood vessel growth [17, 18]. In brief, tamoxifen (Sigma, T5648) was given to *Cdh5-CreERT:mT/mG* and *Apln-CreERT:mT/mG* mice to induce Cre expression. The fluorescence of the mouse retinal vessels was observed by using a fluorescence microscope following the Cre expression. Tamoxifen, diluted in corn oil at 10 mg/ml, 50 μl at 1 mg/ml was injected via intraperitoneal (i.p.) from P2 to P4 for pups, and 200 μl at 10 mg/ml every 2 days, 4 times for adult mice. Different tamoxifen injection time at P5-P7, with P5 as tamoxifen injection at P3-P5, P6 as P4-P6, P7 as P5-P7 and dissected the retina all at P7 to observe the sprouting angiogenesis.

### Separation of retinal endothelial cells

Trypsin digestion was used to separate retinal ECs according to a modified protocol previously published [19]. Briefly, the eyes were enucleated and fixed in 10% neutral buffered formalin for at least 24 h, then equatorially bisected and the entire retinas were removed. The retinas were washed overnight in distilled water and incubated with 3% trypsin (Difco 1:250) in 0.1 M Tris buffer (pH 7.8) at 37 °C with gentle to no shaking. Non-vascular tissues were carefully brushed away following completion of the digestion. Retinal vasculature was then transferred into a 1.5 ml tube containing 450 U/ml Collagenase II (Sigma, V900892), 125 U/ml Collagenase XI (Sigma, C7657), 60 U/ml Hyaluronidase (Sigma, H1115000) in water bath at 37 °C for 1 h to digest retinal vasculature into single cell suspension including ECs, FACS was used to isolate membrane eGFP and non-membrane eGFP cells.

### RNA purification, RT-qPCR and mRNA quantification

Total RNA was extracted by Trizol from eGFP or non-eGFP ECs through FACS sorting of *Apln-CreERT:mT/mG* mouse retina at postnatal day 7 (P7), then reverse transcribed by SuperScript First Strand Synthesis System (Invitrogen) to cDNA. The RNA extracted from the fixed retina cells was performed according to a modified previously published protocol [20, 21]. Total RNA was isolated from fixed cells using reagents from the RNeasy FFPE kit (Qiagen) and the RNeasy Plus Mini Kit (Qiagen), according to a modified version of the vendors’ protocols. Cells were initially re-suspended in 240 μl of Buffer PKD. Following the addition of 10 μl of proteinase K, samples were incubated at 56 °C for 15 min, then at 80 °C for 15 min. 500 μl of Buffer RBC was added and then samples were passed through the gDNA Eliminator column. After the addition of 1200 μl of 100% ethanol to the flow-through, samples were passed through the RNeasy Mini-Elute spin column. Samples were washed with Buffers RW1 and RPE and eluted with RNase-free water following the vendor’s protocol. An on-column DNase digestion was performed as described above for RNA isolation from fresh cells. RNA isolated from sorted cells was used as samples for the probe-based NanoString system and therefore did not undergo DNase treatment. Expression of genes (*Cdh5* and *Apln*) was quantified by RT-qPCR with primers as follows:

*Cdh5*-forward, 5′-CGTGAGCATCCAGGCAGTGGTAGC-3′.

*Cdh5*-reverse, 5′- GAGCCGCCGCCGCAGGAAG-3′.

*Apln*-forward, 5′-ATGAAT CTG AGG CTC TGC GTG CAG-3′.

*Apln*-reverse, 5′- ACT TGG CGA GCC CTT CAATC-3′.

### Retinal sprouting angiogenesis at hypoxia condition

Tamoxifen was injected to pups from P2 to P4 via i.p. as described above. The hypoxia condition was performed according to previous protocol [22] with pups and their dams placed in 75% oxygen till sacrifice to collect the retina for angiogenesis analysis at P7.

### Tumor sprouting angiogenesis in a dorsal skinfold window chamber model

The transparent skinfold window chamber model is an established in vivo system that enables direct visualization of real-time angiogenesis in the tumor. Sprout angiogenesis was examined in the xenograft tumor, as well as in the normal skinfold tissue using two-photon fluorescent microscopy [23–27].

## Results

**Fig. 1:**
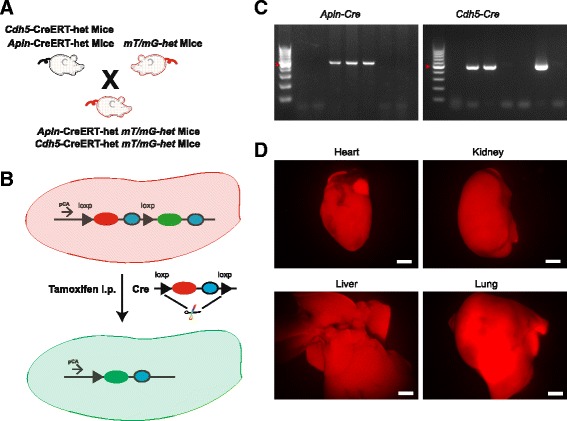
*Apln*-*CreERT*, *Cdh5*-*CreERT* mice, the cell specific membrane (m) fluorescent report mice. **a** The schematic chart illustrates the generation of *CreERT:mT/mG-het* mice. **b** The schematic chart illustrates the mT/mG mouse construct for expression of membrane green eGFP (mG) following tamoxifen administration to remove the red mtdTomato (mT) and stop codon (blue) flanking between 2 *loxP* sites. **c** Genotype bands for *Apln-CreERT* and *Cdh5-CreERT* mice, band for Cre positive and no band for Cre negative mouse. **d** Membrane tdTomato expression in heart, kidney, liver and lung of mT/mG mice. Scale bars: **d**, 2 mm

In *mT/mG* mice, tdTomato-eGFP was inserted in the promoter area of β-actin, and two *LoxP* site were inserted in both ends of tdTomato, therefore without Cre recombinase expression the cells expressed tdTomato protein followed by the stop codon (Fig. 1b, blue oval) and the eGFP protein was not expressed. Following Cre recombinase expression after the tamoxifen injection, the tdTomato sequence and stop codon presented in *LoxP* mice was removed, the EC specific *Cdh5* or *Apln* driven Cre recombinase turned the ECs to express green eGFP protein (Fig. 1a-b). PCR Genotyping results showed that *Cre* was present in the mouse lines, *CreERT* and *mT/mG* double positive mice were used for the experiment (Fig. 1c). Stereoscope observation results confirmed that we had used the right *mT/mG* mice with heart, kidney, liver and lung all expressed tdTomato protein (Fig. 1d).

**Fig. 2:**
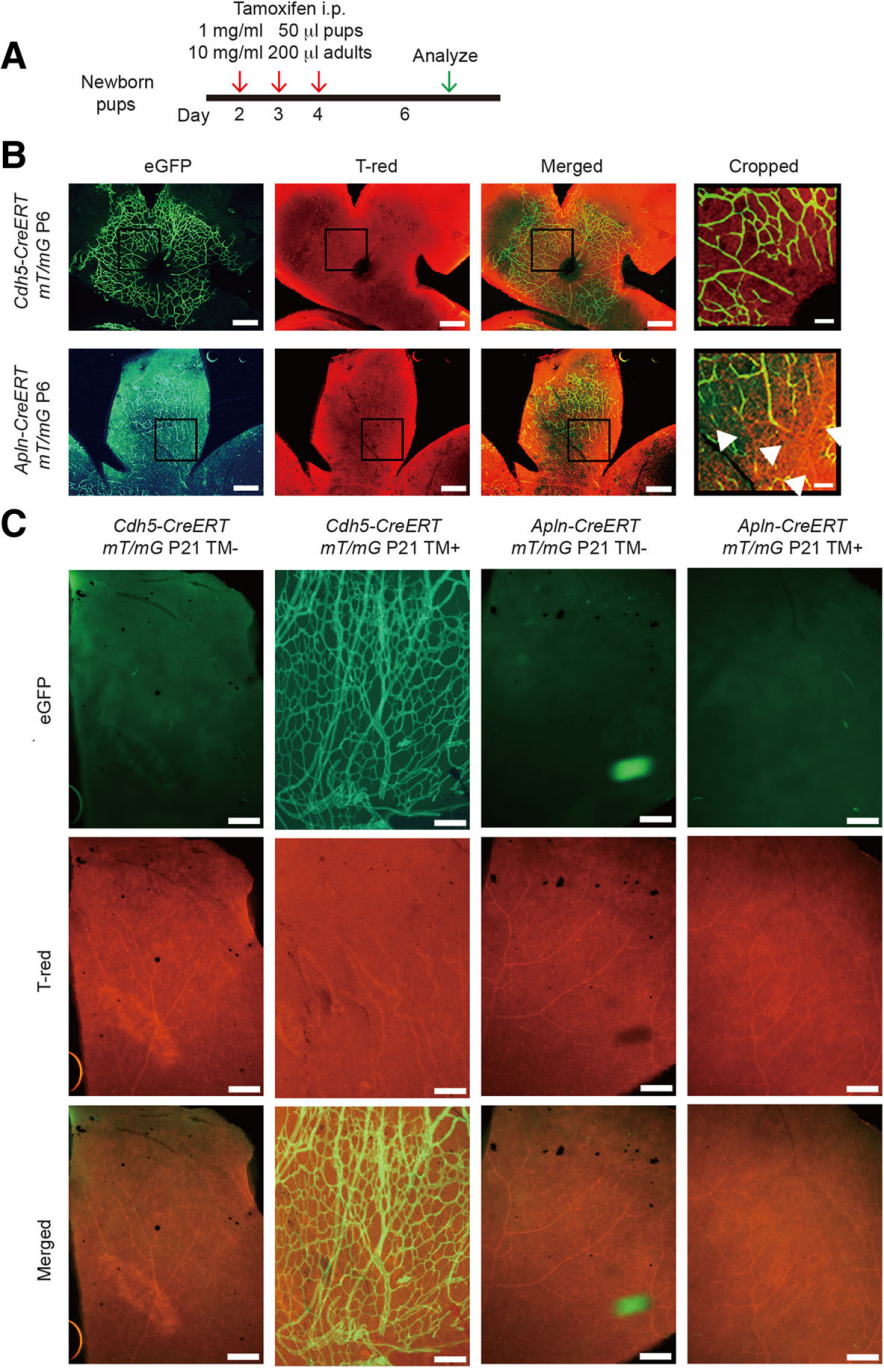
*Apln*-CreERT and *Cdh5-CreERT:mT/mG* mice exhibit different pattern of retina sprouting angiogenesis. **a** The schematic chart illustrates tamoxifen administration for induction of membrane green eGFP expression. **b** *Apln-CreERT:mT/mG* mice exhibit less green-label in the retina “optic nerve” area comparing with that of *Cdh5-CreERT:mT/mG* mice, with green eGFP labeled in the first column, tdTomato labeled in the second column and merged in the third column of the retinal vasculature in *Apln-, Cdh5-CreERT: mT/mG* mice. **c** Adult retina angiogenesis pattern of *Cdh5-CreERT:mT/mG* and *Apln-CreERT:mT/mG* with or without tamoxifen injection. Scale bars: **b**, 500 μm (1st-3rd column), 100 μm (4th column); **c**, 200 μm

Schematic figure showed the strategy for studying the retina angiogenesis of newborn pups by tamoxifen administration in *Cdh5-CreERT:mT/mG* mice and *Apln*-*CreERT:mT/mG* mice (Fig. 2a). Since the superficial vascular plexus forms during the first week after birth by radial outgrowth of vessels from the optic nerve into the periphery [4], so we called the area that close to optic nerve as “optic nerve” area. Comparing retina *of Apln-CreERT:mT/mG* mice with that of *Cdh5*-*CreERT:mT/mG* mice, the “optic nerve” area of *Apln*-*CreERT:mT/mG* mice had less green ECs than ECs of *Cdh5*-*CreERT:mT/mG* mice (Fig. 2b), which might be due to relative mature vessels labeled in *Cdh5-CreERT:mT/mG* mice, but not in *Apln*-*CreERT:mT/mG*. The difference of eGFP signals in the “optic nerve” area between *Apln*-*CreERT:mT/mG* and *Cdh5*-*CreERT:mT/mG* mice suggested that the sprouting angiogenesis can be distinguished from mature angiogenesis vessels using these mouse genetic tools.

In order to see whether the adult retina has similar angiogenesis pattern in these genetic reporter tool mice as retinal developmental angiogenesis model, we examined the adult retina vasculature following tamoxifen administration. The results showed that adult retina of *Apln-CreERT:mT/mG* mice had similar tdTomato fluorescent pattern with no GFP-labeled positive vascular ECs between tamoxifen injection or not injection group, while all ECs in vasculature were GFP-labeled positive in adult retina of *Cdh5-CreERT:mT/mG* mice when administration of tamoxifen, but not in the non-tamoxifen administration group (Fig. 2c). These results suggested that *Cdh5-CreERT:mT/mG* mice could display both mature and sprouting vasculature, while *Apln-CreERT:mT/mG* display sprouting angiogenesis more specifically, which was confirmed by increased retinal sprouting angiogenesis during hypoxia condition when Apelin expression level increased as described below.

**Fig. 3:**
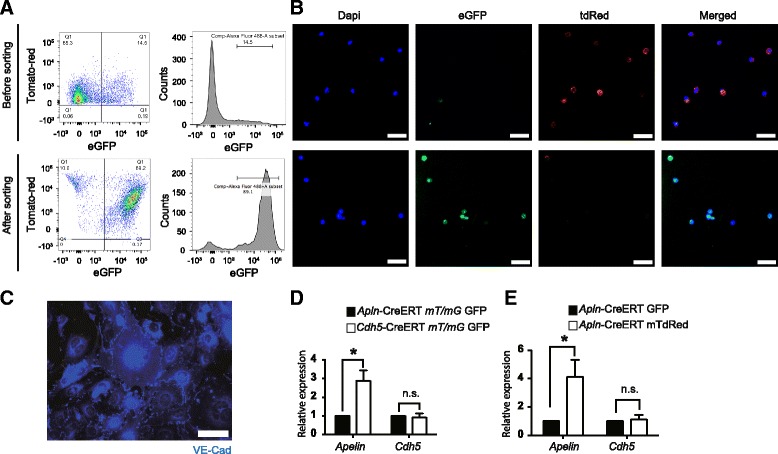
Fluorescence-activated cell sorting (FCAS) separates endothelial cells from *Apln-CreERT:mT/mG* and *Cdh5-CreERT:mT/mG* mice and higher *apelin* expression in GFP positive endothelial cells of *Apln-CreERT:mT/mG* mice. **a** The scatter dot plot (left) and histogram (right) of FACS analysis before and after FACS sorting of green meGFP endothelial cells. **b** ECs before and after FACS sorting under the fluorescent microscope. **c** The FACS sorted ECs are VE-Cadherin positive. **d** *Apelin* mRNA expression in mGFP positive endothelial cells from *Apln-CreERT:mT/mG* is significantly higher than the cells from *Cdh5-CreERT:mT/mG* mice. **e** *Apelin* expression in eGFP positive endothelial cells is significantly higher than tdTomato endothelial cells of *Apln-CreERT: mT/mG* mice. Data quantification was mean ± S.E.M (*n* = 6). All data were analyzed using Student’s t-test unless otherwise noted. n.s., not significant; *, *P* < 0.05. Scale bars: **b**, 100 μm; **c**, 50 μm

Further we separated the *Apln-CreERT:mT/mG* retinal ECs by FACS sorting the single cell suspension of the enzymatic digested retina. The percentage of green eGFP expressing ECs was 14.5% before sorting, reached 89.2% after the sorting (Fig. 3a). This significant increase of eGFP expression cells after FACS was confirmed by fluorescence microscope observation (Fig. 3b), and these cells were also VE-Cadherin positive (Fig. 3c), suggesting the sorted cells were ECs. Quantified PCR (qPCR) showed 2.8-fold higher *Apelin* expression in mGFP ECs of *Apln-CreERT:mT/mG* mice than ECs of *Cdh5-CreERT:mT/mG* mice, while there was no significant difference of *Cdh5* expression in ECs between these two mouse lines (Fig. 3d), suggesting that eGFP ECs in *Cdh5-CreERT:mT/mG* mice contained non-sprouting angiogenetic cells, while *Apelin* is a specific molecular marker of sprouting angiogenesis. Further study showed 3.9-fold higher *Apelin* expression in eGFP ECs than tdTomato ECs of *Apln-CreERT:mT/mG* mice, with no significant difference of *Cdh5* expression between eGFP ECs and tdTomato ECs (Fig. 3e), suggesting that the sorted higher *Apelin* eGFP ECs in *Apln-CreERT:mT/mG* mice represented the sprouting angiogenesis ECs.

**Fig. 4:**
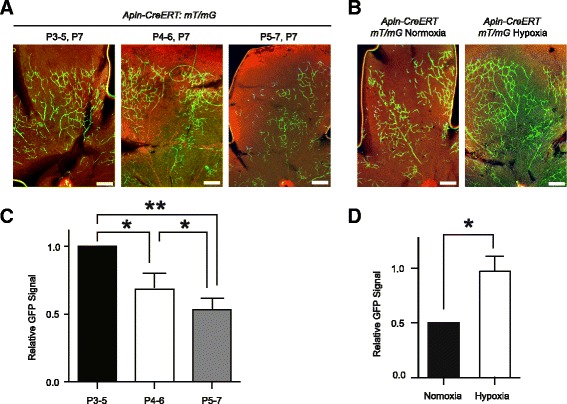
Less retina sprouting angiogenesis in *Apln-CreERT:mT/mG* mice when induced at late postnatal time and more at hypoxia condition. **a-b** The GFP-labeled retina sprouting angiogenesis decreases gradually in *Apln-CreERT:mT/mG* mice following tamoxifen administration from P5-P7 (**a**) with quantification results (**b**). **c-d** *Apln-CreERT:mT/mG* mice exhibit more abundant GFP-labeled retina sprouting angiogenesis at hypoxia than normoxia condition (**c**) with quantification results (**d**). Data quantification was mean ± S.E.M (*n* = 3). All data were analyzed using Student’s t-test unless otherwise noted. n.s., not significant; *, *P* < 0.05; **, *P* < 0.01. Scale bars: **a, b**, 1000 μm

The mouse retinal vessels were formed at the first week after birth with growth of angiogenic sprouting from center to the peripheral, thus we hypothesized different retinal sprouting angiogenesis pattern will be seen following tamoxifen administration at different time during retinal vasculature development. As expected, we found that GFP-labeled sprout angiogenesis ECs decreased gradually when tamoxifen administration from P5-P7 (Fig. 4a, c).

All eukaryotic organisms rely on oxygen (O_2_) to support oxidative phosphorylation for efficient adenosine triphosphate (ATP) production and maintain cell function. Vascular dysfunction due to vessel occlusion or rupture can cause decreased O_2_ delivery, hypoxia, which is a pathogenic driver in diabetic retinopathy [28]. In contrast, rapid cell division during tumor can enhance O_2_ demand due to increasing metabolism and cause localized hypoxia [29]. Hypoxia play a critical role in the pathogenesis of a broad array of disease especially those in which the vasculature is a component, therefore we used the *Apln-CreERT:mT/mG* mice to observe the sprouting angiogenesis pattern in hypoxia retina and tumor model, providing evidence for future molecular mechanisms study of sprout angiogenesis and find therapeutic target for angiogenesis-related diseases.

**Fig. 5:**
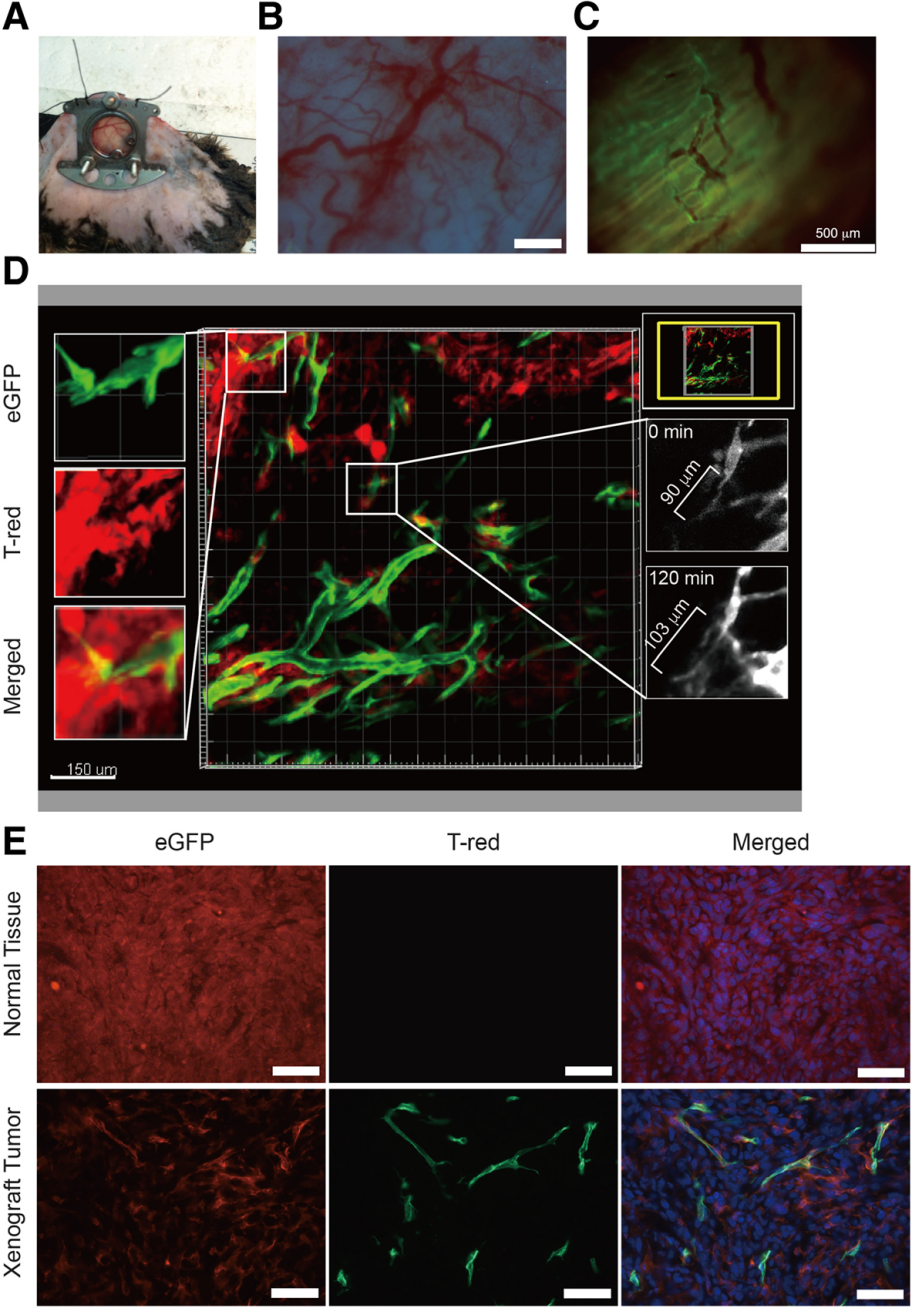
*Apln-CreERT:mT/mG* mice exhibit more tumor real-time sprouting angiogenesis in vivo. **a** A representative image of dorsal skinfold window chamber tumor sprouting angiogenesis model. **b-c** Tumor vessels can be visualized under fluorescent microscope. **d** Dynamic observation of the growth of tumor sprout angiogenesis under two-photon microscope. **e** The vasculature of tumor section and skinfold normal tissue under fluorescent microscope. Scale bars: **b-c**, 500 μm; **d**, 150 μm; **e**, 100 μm

We found that *Apln-CreERT:mT/mG* mice displayed more abundant GFP-labeled retinal sprouting angiogenesis during hypoxia than normoxia condition (Fig. 4b, d). Moreover we used an established dorsal skinfold window chamber model [25] to examine the in vivo in real-time tumor sprouting angiogenesis under two-photon microscope in *Apln-CreERT:mT/mG* mice, we observed green eGFP sprouting angiogenesis in the tumor vasculature, but not in the normal skinfold tissue (Fig. 5a-c). The dynamic observation of tumor vessel sprouting enabled us to measure the sprouting length from 90 μm to 120 μm during two-hours observation period (Fig. 5d). Finally, the xenograft tumor was dissected from the chamber and GFP-labeled sprouting angiogenesis could be seen, but not in normal skinfold tissue (Fig. 5e).

## Discussion

Angiogenesis is a critical progress involving organ development and many angiogenesis-related diseases. Sprouting angiogenesis is the leading stage for the angiogenesis. Better understanding the mechanisms will have tremendous benefit for angiogenesis-related disease therapy. If sprouting angiogenesis phase can be seen in the very beginning, then this will be a useful tool for visualization of direct inhibition of EC proliferation and angiogenesis progression, providing evidence for future intervention of angiogenesis-related diseases.

In this study, we found that *Apln-CreERT:mT/mG* reporter mouse line is a useful tool for evaluation of sprouting angiogenesis during mouse retina vasculature development, in which the high-resolution retinal vasculature imaging could be visualized with easy quantification of sprouting angiogenesis. Less green eGFP ECs in retina “optic nerve” area observed in *Apln-CreERT:mT/mG* mice than in *Cdh5*-*CreERT:mT/mG* mice suggested that *Apln-CreERT:mT/mG* mice displayed sprouting angiogenesis specifically, while sprouting and mature vasculature ECs cannot be distinguished in *Cdh5-CreERT:mT/mG* mice. And these ECs of sprouting angiogenesis can be separated by FACS and we confirmed higher *Apln* expression level in the angiogenic sprouting ECs. These results indicated that *Apln-CreERT:mT/mG* mice can be used to directly visualize the sprouting angiogenesis in vivo vasculature, and the angiogenic sprouting ECs separated by FACS can be used for further mechanism studies.

*Apln-CreERT:mT/mG* mice displayed sprouting angiogenesis during retinal vasculature development. Hypoxia condition is the pathogenic driver for pathologic angiogenesis. As expected, we found that *Apln-CreERT:mT/mG* mice did exhibit more abundant GFP-labeled sprouting angiogenesis at hypoxia than normoxia condition during retinal vasculature development. Since ischemia-induced hypoxia is a major component of several blinding retinopathies [22], this mouse model can be used to examine the effects of small molecules, drugs, even siRNA or viral gene vector infection on in vivo sprouting angiogenesis at hypoxia condition and find appropriate therapy for the retinopathies [30].

Tumor growth is always accompanied by neovascularization, which has been well studied as the therapeutic target [13]. More sprouting angiogenesis occurs during tumor growth and we used two-photon microscope to observe the real-time dynamic sprouting angiogenesis in vivo, which enable us to measure angiogeneic sprouting length. Using this tool, we can quantify the sprouting angiogenesis more accurately and it might be used in the future to screen anti-angiogenic medication to impair the tumor growth.

The tumor angiogenesis is a complicated process and involves in many signal pathways, *Apln-CreERT:mT/mG* mice can be crossed with any gene loss-of-function or gain-of-function mice to study their function on sprouting angiogenesis, and the sprouting angiogenesis ECs can be separated by FACS sorting for detailed cellular function and molecular biology study to find more efficient and less drug resistant medication for therapy of angiogenesis-related diseases as cancer and retinopathy [31].

## Conclusions

We concluded that *Apln-CreERT:mT/mG* mouse is a useful tool that can be used for future in-depth study of sprout angiogenesis in vivo especially in the disease model, such as tumor, diabetic retinopathy to better understand the underlying mechanisms for further therapy.

## Abbreviations

Apln: Apelin
Cre-ERT: Cre recombinase-Tamoxifen induced estrogen receptor
EC: Endothelial cell
FACS: Fluorescence-activated cell sorting
mG: membrane Green Fluorescent Protein
mT: membrane Tomato red
mTdRed: membrane Tomato Red
